# Semantic Segmentation in Large-Size Orthomosaics to Detect the Vegetation Area in *Opuntia* spp. Crop

**DOI:** 10.3390/jimaging10080187

**Published:** 2024-08-01

**Authors:** Arturo Duarte-Rangel, César Camacho-Bello, Eduardo Cornejo-Velazquez, Mireya Clavel-Maqueda

**Affiliations:** 1 Artificial Intelligence Laboratory, Universidad Politécnica de Tulancingo, Tulancingo 43629, Hidalgo, Mexico; arturo.duarte1931041@upt.edu.mx; 2 Research Center on Technology of Information and Systems, Universidad Autónoma del Estado de Hidalgo, Mineral de la Reforma 42184, Hidalgo, Mexico; ecornejo@uaeh.edu.mx (E.C.-V.); mclavel@uaeh.edu.mx (M.C.-M.)

**Keywords:** artificial intelligence in agriculture, precision agriculture, vegetation detection, analysis of aerial images, crop monitoring, drones in agriculture

## Abstract

This study focuses on semantic segmentation in crop *Opuntia* spp. orthomosaics; this is a significant challenge due to the inherent variability in the captured images. Manual measurement of *Opuntia* spp. vegetation areas can be slow and inefficient, highlighting the need for more advanced and accurate methods. For this reason, we propose to use deep learning techniques to provide a more precise and efficient measurement of the vegetation area. Our research focuses on the unique difficulties posed by segmenting high-resolution images exceeding 2000 pixels, a common problem in generating orthomosaics for agricultural monitoring. The research was carried out on a *Opuntia* spp. cultivation located in the agricultural region of Tulancingo, Hidalgo, Mexico. The images used in this study were obtained by drones and processed using advanced semantic segmentation architectures, including DeepLabV3+, UNet, and UNet Style Xception. The results offer a comparative analysis of the performance of these architectures in the semantic segmentation of *Opuntia* spp., thus contributing to the development and improvement of crop analysis techniques based on deep learning. This work sets a precedent for future research applying deep learning techniques in agriculture.

## 1. Introduction

The United Nations 2030 Agenda establishes a global imperative to reconfigure our society, guided by 17 Sustainable Development Goals (SDGs). These goals, from poverty eradication to sustainable agricultural practices, outline a path toward a more equitable and resilient future. In particular, SDG 2 emphasizes the need to eradicate hunger, ensure food availability, improve nutrition, and promote sustainable agriculture [1]. This goal highlights the importance of implementing productive and sustainable agricultural practices resilient to climate variations. In this framework, the *Opuntia* spp. (nopal) emerges as a paradigmatic crop. Native to Mexico and prevalent in arid and semi-arid regions, this *Opuntia* spp. is an essential source of nutrition for many communities and symbolizes resilience and adaptability in the face of climate challenges [2], making its sustainable cultivation crucial in feeding countless people and preserving vulnerable ecosystems.

The cultivation of *Opuntia* spp. stands out for its resistance to adverse conditions and its versatility in food and industry; its cultivation is a traditional practice in several regions, especially in Mexico, thanks to its multiple uses and benefits [3]. *Opuntia* spp. are essential as food, fodder, and industrial products; they play a crucial role in soil and water conservation, carbon fixation, and biodiversity [4]. With the growing interest in products derived from *Opuntia* spp., farmers and researchers seek to optimize their production and management.

In 2022, in Mexico, the annual per capita consumption of *Opuntia* spp. was 6.2 kg. A total of 12,491 hectares were cultivated, with a production of 872 thousand tons and an average yield of 70.5 tons per hectare, generating a value of MXN 2981 million (USD 175.3 million) at an average price of MXN 3417 per ton. Additionally, 7.4% of the production was exported, reaching a value of USD 26.2 million [5].

In agriculture, precision and efficiency in measuring vegetation area are crucial for optimal crop management. Although *Opuntia* spp. cultivation is known for its resistance and versatility, its effective management requires precise monitoring. Traditional manual techniques, while effective, are laborious and prone to errors. In contrast, emerging technologies such as remote sensing and artificial intelligence offer promising solutions to overcome these limitations. Integrating high-resolution orthophotos with advanced semantic segmentation techniques promises to revolutionize how we measure and manage vegetation area in *Opuntia* spp. cultivation. This research seeks to explore and validate the effectiveness of these advanced techniques, specifically in the agricultural region of Tulancingo, Hidalgo, Mexico.

The integration of advanced technologies allows agricultural practices to be adapted to the specific conditions of each plot, thus optimizing resource use and improving productivity. Remote sensing, an essential tool for this approach, facilitates the monitoring of the health and condition of vegetation through remote sensors.

Precision agriculture (PA) has been established as a promising solution for increasing crop efficiency and productivity. Based on observation, measurement, and action on intra-field variability, precision agriculture seeks to optimize crop yields and minimize production costs [6].

Using platforms such as satellites, airplanes, and unmanned aerial vehicles (UAVs), remote sensing has proven a valuable tool in quantifying vegetation for precision agriculture [7,8]. These sensors collect data in various spectral bands, allowing detailed analysis of plant health, water content, and plant density, among other critical factors [9].

In viticulture, for example, developing specific vegetation index has revolutionized how we monitor vineyard health and optimize vineyard management [8]; with the data, winegrowers can make informed decisions about critical practices such as irrigation, fertilization, and pest control.

A study by Sebastian Candiago et al. [10] highlights the value of UAVs for rapidly collecting field data in precision agriculture applications. Equipped with multispectral cameras, these UAVs produce high-resolution images that, once processed, result in precise orthoimages of the studied areas. Such orthoimages facilitate the extraction of vegetation indices (VI) that evaluate the vigor of vegetation in various crops, thus demonstrating the considerable potential of high-resolution data from UAVs and photogrammetric techniques in agriculture.

Furthermore, integrating machine learning techniques with remote sensing has significantly strengthened the ability to predict and manage agronomic aspects with greater precision [11]. These modern methods, in conjunction with data obtained from remote sensors, provide a more detailed and quantitative perspective of agricultural fields, facilitating more effective and sustainable management of agriculture.

Advanced hyperspectral technologies have enabled innovative studies and practical applications in agriculture, ranging from regional scales through satellite platforms to the field level, using airplanes, drones, and ground platforms [12]. Integrating AI into these systems has further revolutionized the field of remote sensing and smart agriculture, opening new avenues to optimize agricultural production.

Moreover, the combination of AI and remote sensing has emerged as an effective technological solution to support the improvement of agricultural yields, offering advanced methods for data analysis to increase productivity, anticipate possible threats, and alleviate the workload of farmers [13]. In this environment, image analysis has become an invaluable tool, allowing farmers to access detailed information about their crops and supporting them to make data-driven decisions.

The application of advanced technologies in agriculture, as demonstrated in recent studies on broccoli growth monitoring, underlines the importance of adaptability and precision in crop management [14]. The present *Opuntia* spp. study seeks to expand the application of these technologies, demonstrating how the integration of UAV imagery and deep learning can optimize precision agriculture for specific crops. The ability to accurately identify the status and growth of individual plants can revolutionize cactus pear management, enabling a more informed response to environmental variations and improving sustainability and efficiency.

Traditional vegetation segmentation methods, while widely used, present several significant limitations that need to be urgently addressed for more effective solutions in agricultural research. For example, threshold-based methods are susceptible to lighting conditions and plant color and texture variations, resulting in inaccurate segmentations under different environmental conditions [15]. Furthermore, manual segmentation and semi-automatic methods require much time and human effort, making them unfeasible for large crop areas. On the other hand, methods that use specific image characteristics, such as texture and color, may not generalize well to different types of crops and different growth phases of the same plant, limiting their applicability to various agricultural situations [16]. Compared to more advanced deep learning techniques, these methods may not be robust enough to handle the complexities and variability of crops [17]. Although convolutional neural networks (CNNs) have proven to be very effective for image segmentation, they require a significant amount of computational resources for training and inference, including the need for high-end GPUs, which may not be accessible to all farmers [18]. Additionally, CNNs and other deep learning techniques require large volumes of labeled data to train adequately. Collecting and labeling this data can be expensive and time-consuming [19]. Another significant area for improvement is that methods developed in a specific context may not directly apply to other regions with different soil conditions, climates, and crop types. Generalizability is a significant challenge [20]. Furthermore, many studies are conducted in controlled environments or with limited datasets, which may not fully reflect the diversity and complexity of real-world agricultural scenarios [21].

Semantic segmentation, an image processing technique that partitions an image into segments corresponding to objects or specific semantic categories, is particularly relevant in agriculture, which can be used to identify and quantify different elements in a field, such as individual plants, areas affected by diseases or pests, and areas that require irrigation [22,23].

In the realm of semantic segmentation, models such as DeepLabV3+ [24], UNet [25], and UNet Style Xception [26] have proven to be highly effective, not only in medical and agricultural applications, thanks to its ability to capture characteristics at different scales. These models generally combine convolutional dilation and decoding techniques to improve segmentation accuracy, and they are widely adopted for their effectiveness.

The advantages of AI and semantic segmentation in agriculture are notable. These technologies provide a detailed and quantitative interpretation of agricultural data, allowing farmers to make decisions based on accurate information [23]. Additionally, image interpretation automation minimizes manual inspections, representing significant savings in time and resources.

Recent research on plant counting using aerial imagery offers a helpful parallel to our study. This approach—initially applied to tobacco crops—combines deep learning techniques and semantic segmentation to count plants from aerial images accurately, thus demonstrating the efficiency of this technology in optimizing agricultural management [27].

Another notable study used UAVs to capture images and applied the UNet Style Xception model to successfully segment the parasitic species Cuscuta spp. in chili tree crops. This method improved the accuracy in identifying affected areas and facilitated the creation of orthomosaics useful for decision making in crop management [28]. Although the results from using UAV data were promising, it is essential to note that the study did not include a validation of these findings. This limitation highlights the need to perform a more thorough validation of the model, encompassing not only individual images but also a broader view of the entire crop to ensure the applicability and accuracy of the method in broader agricultural contexts.

The versatility of these techniques to adapt to different crop types highlights their potential to improve accuracy in measuring and managing vegetation areas, a crucial aspect of precision agriculture. In particular, precision agriculture can help identify areas of optimal growth, optimize resource use, and improve both the quality and quantity of production for *Opuntia* spp. cultivation. In this context, the present study contributes significantly to advancing precision agriculture. The main contributions of this research include:

- Detailed comparison: A comprehensive comparison of the performance of different semantic segmentation architectures is provided, including DeepLabV3+, UNet, and UNet Style Xception.
- Validation in a specific context: The research validates the effectiveness of advanced deep learning techniques in the particular context of the agricultural region of Tulancingo, Hidalgo, Mexico.
- Optimization of vegetation measurement: The study proposes a methodology to carry out a more precise and efficient measurement of the *Opuntia* spp. vegetation area, overcoming the limitations of traditional manual techniques.
- Integration of orthophotos and AI: The combination of high-resolution orthophotos obtained by UAV with advanced semantic segmentation techniques stands out.
- Contribution to crop analysis techniques: This study significantly contributes to developing and improving deep-learning-based crop analysis techniques.

One of the main innovations of this work lies in overcoming the difficulty of segmenting images with resolutions greater than 2000 pixels, especially in orthomosaics, using well-known segmentation methods. This is a significant contribution, given that no studies or previous methods have addressed this problem. Likewise, we highlight the need for manual quantification for pixel counting, given that current neural networks have limitations in handling extremely large images; this aspect is crucial to guarantee precision in measuring the area of pixels and vegetation and underlines the relevance of our proposed methods. The advances presented not only improve the accuracy and efficiency of vegetation analysis but also open new possibilities for agricultural management and monitoring.

With these contributions, a technological tool is presented to carry out measurements that facilitate the analysis, measurement, and management of the vegetation area in *Opuntia* spp. cultivation to promote a more sustainable and efficient agriculture. The following sections will detail the study area, data acquisition, and the most-used semantic segmentation techniques. In the Section 3, we describe the processing of Orthomosaic images of *Opuntia* spp. cultivation and deep learning techniques to segment areas of interest. In the Section 4, we present a comparative analysis of the architectures in semantic segmentation and the measurement of the *Opuntia* spp. vegetation area. Finally, we report the conclusions of the study.

## 2. Materials and Methods

### 2.1. Study Area

**Figure 1:**
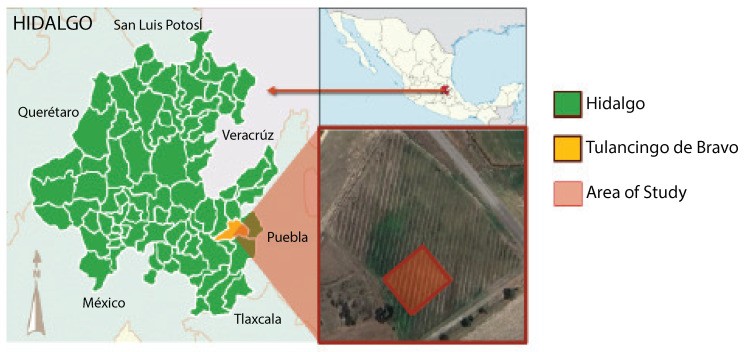
Geographic location of the study area.

The object of study focuses on a nopal cultivation (*Opuntia* spp.) located at the geographical coordinates 20.12555 latitude and −98.37867 longitude, in the municipality of Tulancingo, state of Hidalgo, Mexico. The exact location is illustrated in Figure 1. The crop field is located near the Tulancingo–Huauchinango highway, approximately 1 km from the Polytechnic University of Tulancingo.

The investigation focuses on a specific crop area comprising around 539 m^2^. This delimitation is established to have a representative sample that enables an exhaustive evaluation of data acquisition and processing techniques.

Accessibility and proximity to the laboratory justify the location selection, thus optimizing the logistics for data collection and subsequent analysis. Furthermore, this proximity allows for a detailed characterization of soil, vegetation, and other relevant elements. Therefore, choosing a representative area ensures the research findings significantly impact *Opuntia* spp. crops in the Tulancingo region.

### 2.2. Data Acquisition

Meticulous and detailed data acquisition is essential to thoroughly understanding the development of *Opuntia* spp. cultivation from its initial stage to maturity. This process allows us to capture the crop’s physical evolution and identify the management practices that optimize its growth and health. In practice, the stems are arranged in central furrows during planting, facilitating their growth and management. As the stems take root, they develop shoots that become new cladodes. Furthermore, the technical management of *Opuntia* spp. cultivation involves various field practices, mainly focused on maintaining the health of the plantation; this includes pruning, sprouting, and rejuvenation training to improve the quality and quantity of products available for the market [29,30]. All considerations mentioned are essential for the success of *Opuntia* spp. cultivation in several regions, including Tulancingo.

Integrating advanced technology in this process emphasizes choosing tools and technologies that allow processes to be carried out with maximum efficiency and precision, considering the ability to capture fine details of the crop and its adaptability to specific field conditions. We used the DJI Mavic 2 Mini (DJI, Shenzhen, China) drone for data acquisition, which weighs just 249 g. The drone is equipped with a 1/2.3″ 12-megapixel CMOS image sensor, capable of capturing high-quality images. Its compact design is ideal for flights near structures and vegetation. Plus, it features multiple sensors, including GPS, for precise navigation; the battery offers up to 25 min of continuous flight, while its intuitive remote control and Dronelink mobile app (Dronelink, Austin, TX, USA) simplify flight mission planning and execution.

**Figure 2:**
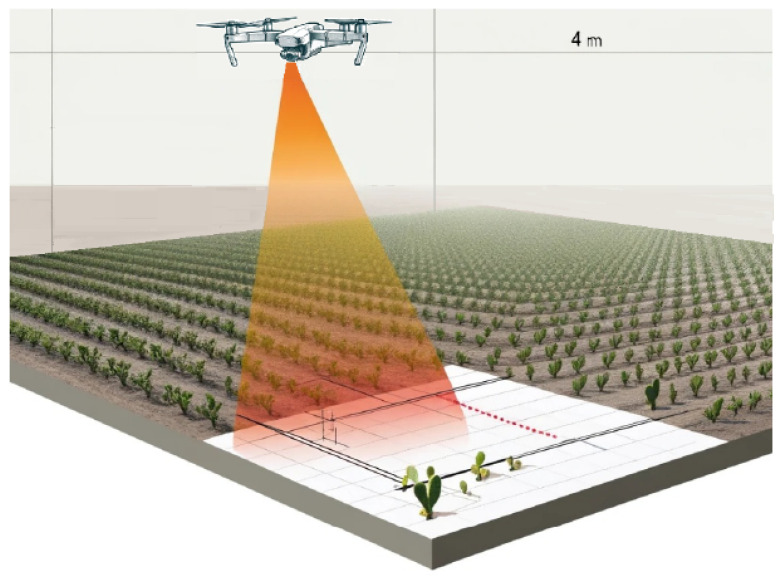
Flight plan at 4 m height.

Combining high resolution with maneuverability and adaptability becomes essential in research methodology. The flight plan is meticulously executed over the area of interest at a height of 4 m, with the primary objective of evaluating and validating the robustness of the semantic segmentation models used in this research. The chosen height significantly improves the quality and reliability of the results obtained in the study. Figure 2 shows a diagram of the flight carried out over the crop.

Precise flight trajectory plans are designed with Dronelink software (Version 3.4.0) with 75% lateral and 70% vertical overlap, ensuring comprehensive coverage of the area of interest. All flights take place around 11:00 a.m. to ensure stable lighting conditions.

Furthermore, three flight missions are carried out with different configurations to capture images from various angles and perspectives. Settings include a vertical orientation of 90° directly above the crop, a tilt of 45° to obtain lateral images of the crop, and a tilt of 135° to capture a more perspective and facilitate a more complete and detailed evaluation of the *Opuntia* spp. crop, adequately documenting all relevant characteristics. During the flights, 443 high-resolution aerial photographs were taken. Table 1 details the images captured for each flight.

### 2.3. Semantic Segmentation

Semantic segmentation constitutes one of the main techniques in image analysis, allowing the identification and classification of each pixel according to predefined semantic categories. This approach is particularly valuable in photogrammetric studies aimed at distinguishing between different features and objects present in aerial images, especially in applications related to managing and exploiting plantations. In the present research, leading architectures in semantic segmentation are implemented, such as UNet, DeepLabV3+, and UNet Style Xception. The following subsections concisely describe these architectures, highlighting their contributions and applicability in semantic segmentation.

#### 2.3.1. UNet

**Figure 3:**
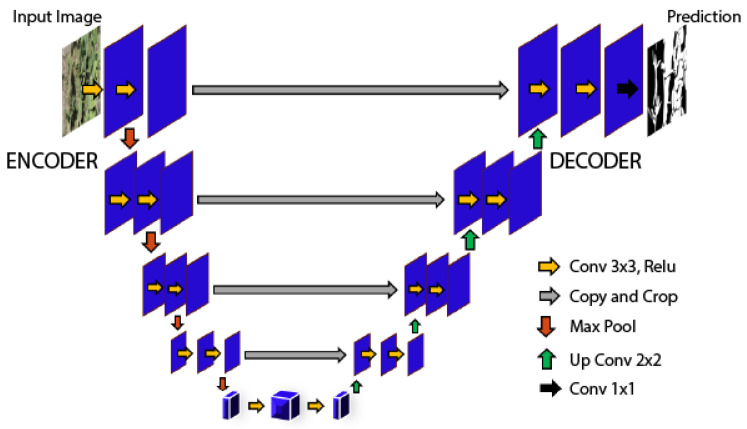
UNet structure for the segmentation of the *Opuntia* spp. vegetation area.

The UNet architecture for segmentation has its characteristic “U”-shaped design, integrating encoding and decoding sections to process and reconstruct images with high precision [25]. It excels at detecting fine details, which are crucial for identifying affected crop areas. Its distinctive design, depicted in Figure 3, is based on a symmetric topology with an encoder (contraction path) and a decoder (expansion path) connected by a central bridge.

The encoder is composed of repetitive blocks of two 3×3 convolutions followed by a ReLU (rectified linear unit) activation function and a 2×2 max pooling operation with stride 2, which progressively reduces the dimensionality of the feature space, allowing the network to learn more abstract and robust representations of the input data.

In contrast, the decoder is structured by blocks of 2×2 up-convolutions that increase the resolution of the mapped features, followed by concatenations with the corresponding encoder features, technically called “copy and trim”. This process recovers location information lost during clustering and combines it with higher-level features to facilitate accurate segmentation.

Finally, each expansive step is completed with two 3×3 convolutions and ReLU, and the sequence culminates in a 1×1 convolution layer that maps the high-dimensional features to the desired classification per pixel, resulting in the segmentation prediction image.

The strength of UNet lies in its ability to operate with a limited number of training samples and still produce highly accurate segmentations, highlighting its usefulness in applications where data can be sparse or extremely varied.

#### 2.3.2. DeepLabV3+

**Figure 4:**
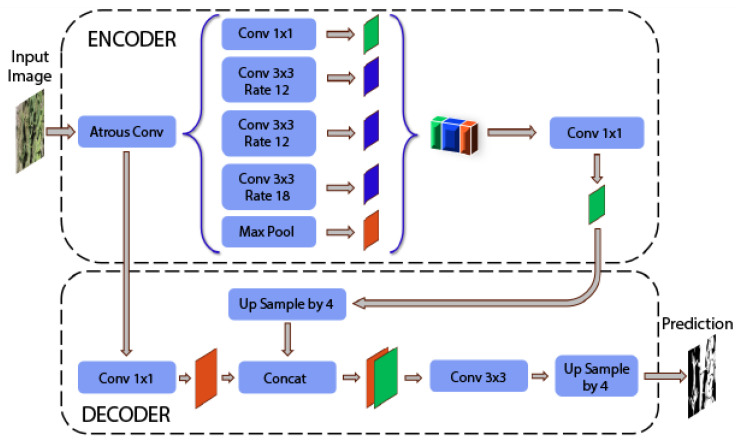
Structure of DeepLabV3+ for segmenting the *Opuntia* spp. vegetation area.

The DeepLabV3+ [24] architecture, illustrated in Figure 4, is a significant innovation for semantic segmentation, designed to improve accuracy at object edges, and in small-scale regions. This network expands the context capture capability and enhances the contour accuracy by implementing atrous convolution modules in its encoder and decoder structure.

The DeepLabV3+ encoder starts with an atrous convolution that adjusts the network’s field of view to the image features and continues with parallel atrous convolution blocks with different dilation rates (ratios), allowing the network to capture contexts in multiple scales without losing image resolution. These atrous convolutions, also known as dilated convolutions, process the image at various spatial scales and allow you to explicitly control the resolution at which the feature operation is carried out, preserving the edge information of the objects.

The decoder uses ‘upsampling’ operations to increase the resolution of the extracted features. These extended features are concatenated with the encoder’s high-level features through a ‘concatenation’ procedure to preserve detailed image information. Then, a series of 3×3 convolutions are applied to refine the combined features, followed by a second ‘upsampling’ operation to achieve the resolution of the original image.

Finally, the architecture applies a 1×1 convolution to obtain the segmentation prediction. This last layer projects the multidimensional feature tensor into the segmentation label space, producing the final prediction image.

The architecture is distinguished by its ability to handle objects of various scales and its efficiency in precisely segmenting fine and complex details, as required in detecting specific areas of vegetation in *Opuntia* spp., making it a preferred option for applications that demand high fidelity in segmentation.

#### 2.3.3. UNet Style Xception

In the development of advanced techniques for image segmentation, the UNet Style Xception architecture is presented as a significant innovation. Combining UNet with the Xception architecture, using separable convolutions [31], results in a highly efficient and accurate system that improves the network’s encoding, decoding, and feature detection. The original UNet is characterized by its contraction path, which captures the overall context of the image, and an expansion path that reconstructs the segmentation output from those encoded features. Xception, for its part, introduces separable convolution modules, which allow more efficient handling of spatial and channel characteristics.

In the contraction path, the Xception-Style architecture improves context capture by efficiently processing low- and high-dimensional features. This results in an improved ability to identify and encode relevant image information. On the other hand, the expansion path benefits from the accuracy of separable convolutions, which leads to a more accurate reconstruction of the segmented image. Additionally, feature detection is refined, allowing finer segmentation and greater model generalization.

**Figure 5:**
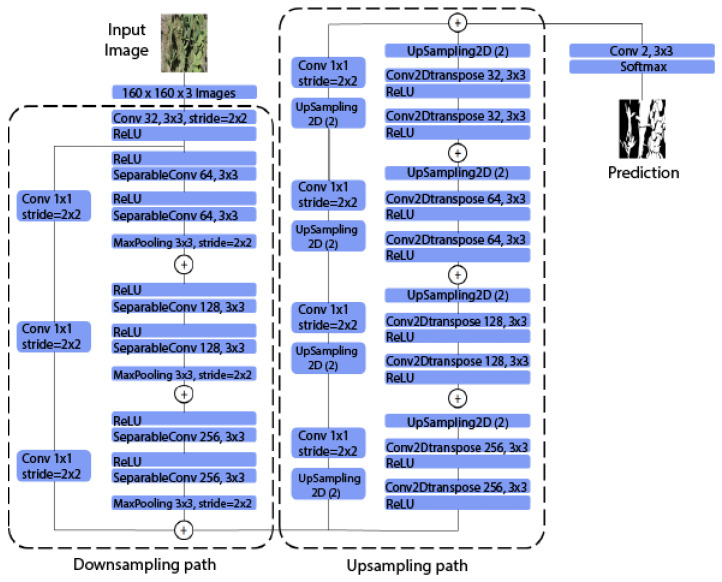
Structure of the UNet Style Xception for segmenting the *Opuntia* spp. vegetation area.

The Xception architecture indicates that its integration with UNet is theoretically sound and empirically validated. Such fusion expands the capabilities of the UNet convolutional neural network and inaugurates new possibilities in remote sensing and geospatial analysis. Figure 5 exposes the structure of UNet Style Xception in the segmentation of *Opuntia* spp. vegetation, emphasizing its effectiveness in the face of the complexity of aerial images.

### 2.4. Generation of Orthomosaics

Implementing advanced technologies has emerged as a fundamental pillar for optimizing crop production and management in agriculture. One of these technologies is the generation of orthomosaics, which has transformed the perception and understanding of agricultural land.

Among these innovations, orthomosaics have emerged as a transformative tool that revolutionizes how farmers visualize and understand their farmland. Orthomosaics are the result of processing and integrating aerial images, offering a detailed and panoramic perspective of the terrain, thus allowing informed and accurate decision making.

An orthomosaic is a georeferenced and orthorectified image, the result of the integration of multiple aerial photographs. Mathematically, orthorectification can be described as a transformation *T* that corrects the geometric distortions of an image *I* to obtain a planar representation $I^{\prime }$:

(1) $$\begin{array}{c} I^{\prime }=T\left(I\right) \end{array}$$

The orthomosaic generation process stands out for its structure and precision, covering several essential stages to produce a high-quality final result. Initially, we capture images using aerial platforms like drones. Aerial photographs ensure adequate overlap on the *x* and *y* axes, thus ensuring complete terrain coverage.

After capturing, we proceed to image processing. This step is characterized by meticulous alignment and correction, where key point-matching algorithms identify similar regions between neighboring images. In this process, tools such as WebODM (Version 2.5.0, Open Drone Map, open-source) software specialized in aerial image processing play a crucial role in facilitating the generation of orthomosaics, 3D models, and elevation maps. These tools’ web-based interface simplifies project management and visualization, while their modular architecture promotes integration with a variety of tools and plugins [32].

**Figure 6:**
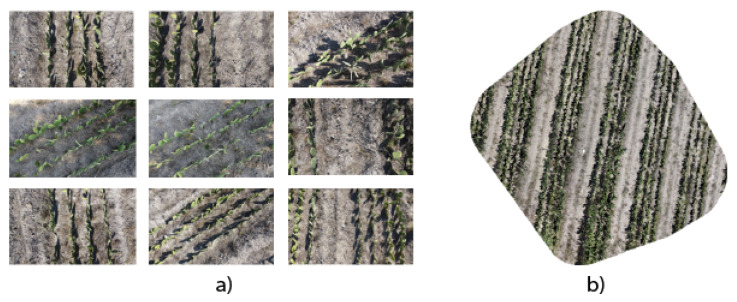
(**a**) Images captured of the study area with the Dji Mavic 2 mini drone; (**b**) orthomosaic generated from the captured images.

Finally, we integrate the georeferenced images to create a continuous orthomosaic. The process eliminates redundancies and ensures a smooth transition between images, producing a cohesive and detailed orthomosaic. Figure 6 illustrates image captures taken over a specific area and the corresponding orthomosaic generated with WebODM.

This example illustrates how we transformed individual aerial images into a unified orthomosaic representation. This transformation provides a detailed, panoramic view of the crop field, enabling applications such as plant health monitoring, crop planning, resource management, and canopy disease detection. In addition, it enables the training of machine learning algorithms to identify growth patterns or detect variations in vegetation. With technological advances and the continuous improvement of aerial image capture and processing techniques, orthomosaics are consolidated as essential tools in modern agriculture.

## 3. Methodology

In the current agricultural landscape, adopting advanced technologies is emerging as an indispensable strategy to face the challenges inherent to efficient and environmentally sustainable production. Remote sensing using UAVs and integrating AI techniques emerge as key tools for optimizing agricultural management. The primary objective of this study is to provide farmers with an advanced analytical tool designed to improve accuracy in the identification and management of vegetation zones, facilitating the implementation of sustainable cultivation practices, and the adoption of optimized agricultural management strategies. The developed methodology is articulated around a detailed process of seven stages, each aiming to maximize the effectiveness of analyzing data obtained through high-resolution images captured by UAVs.

1. Image acquisition via UAV: A flight mission using unmanned aerial vehicles (UAV) is scheduled and conducted to capture high-resolution images of the *Opuntia* spp. cultivation. The mission considers parameters such as altitude, image overlap, and atmospheric conditions to guarantee the quality and precision of the acquired images.
2. Image size reduction: Given the original dimensions of the images captured by the UAV, it is crucial to reduce their size to facilitate and speed up the semantic segmentation process. Reduction must be performed carefully so as not to compromise the quality and essential information of the image.
3. Semantic segmentation: With the reduced images, the corresponding binary masks are obtained using pre-trained semantic segmentation models, such as DeepLabV3+, UNet, and UNet Style Xception, to segment the *Opuntia* spp. areas, differentiating them from the background and other elements present.
4. Mask size augmentation: When obtaining the mask, an up-sizing process is performed to ensure that it has the exact dimensions of the original image and that the segmented areas match the real areas in the crop.
5. Coloring of *Opuntia* spp. areas: The areas identified as *Opuntia* spp. on the masks are colored blue to facilitate analysis and clear distinction between the areas of interest.
6. Orthomosaic generation: The acquired images generate an orthomosaic to offer a panoramic and detailed view of the *Opuntia* spp. cultivation, allowing the farmer to obtain a general perspective of the affected and unaffected areas. A natural extension of this work is the implementation of automatic plant counting algorithms using neural networks or other automatic methods; while the current focus is on semantic segmentation to detect vegetative areas, we are developing additional models that will allow the number of plants directly in segmented images to be counted. The integration will provide a more complete and accurate crop measurement and improve agricultural management efficiency by automating crucial monitoring tasks.
7. Quantification and analysis: Finally, using the generated masks and the orthomosaic, a quantitative analysis is carried out, determining the total extent of the vegetation area and providing precise metrics to guide agricultural decisions and necessary interventions.

**Figure 7:**
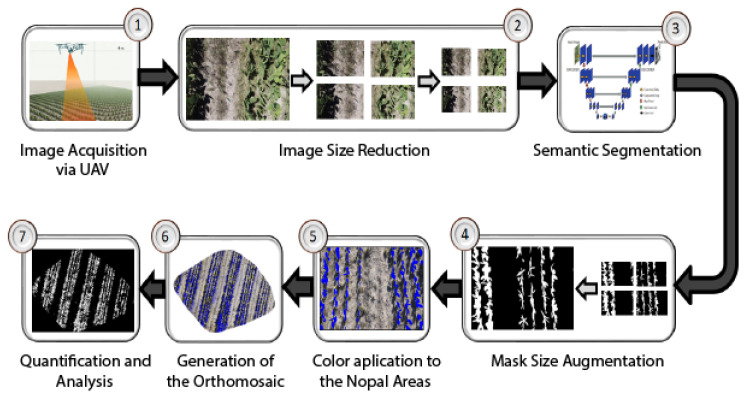
Step diagram of the proposed methodology for quantifying *Opuntia* spp. vegetation area.

The proposed methodology seeks to provide farmers with precise and practical tools for managing, exploiting, and maintaining fields with *Opuntia* spp. cultivation. Combining high-quality images with advanced image processing techniques and AI guarantees reliable and actionable results. Figure 7 shows a diagram of the main steps of the proposed method.

### 3.1. Dataset Generation

Constructing a robust and representative dataset was crucial for developing the model. Given the high computational cost associated with processing high-resolution images, we adopted an efficient methodology based on reducing the size of the images before their introduction into the model, following the recommendations of Gutiérrez-Lazcano et al. [28].

The process begins with acquiring original images with dimensions of 4000×2250 pixels. Each image was divided into four equal segments of 2000×1125 pixels, aiming to more efficiently manage computational resources without sacrificing data integrity. Subsequently, these segments were scaled to a uniform dimension of 160×160 pixels to facilitate memory management during model training and ensure processing speed.

**Figure 8:**
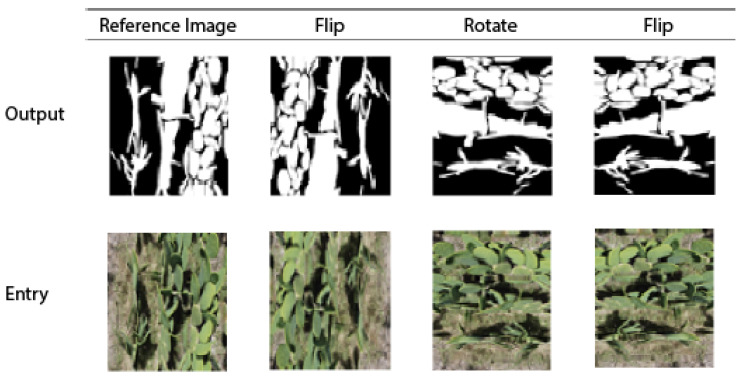
Image transformations for data augmentation.

A data augmentation scheme is implemented on the scaled images to increase the diversity and data available for training. The augmentation includes transformations such as flip and 90- and 180-degree rotation. Figure 8 shows some examples of transformations.

From the 294 images taken from the three flights as training images (Table 1), with augmentation and division into four images, we have 4704 images. Data augmentation techniques are essential for training robust AI models, as they allow the model to learn from a wider variety of perspectives and conditions, improving its generalization ability and performance on previously unseen data.

### 3.2. Application of Deep Learning Techniques

The efficient application of deep learning techniques in semantic segmentation entails a set of stages, from the capture and adaptation of the data to the refinement and evaluation of the generated models. This work uses deep learning models, specifically DeepLabV3+, UNet, and UNet Style Xception. The models are selected for their demonstrated effectiveness in semantic segmentation tasks and their easy adaptability to identify and analyze *Opuntia* spp. vegetation within high-resolution images acquired by UAVs.

The models were implemented in Python 3.8, using TensorFlow 2.5 within PyCharm as an IDE to facilitate building and tuning the models on a Windows 10 system. The hardware includes an Intel Core i7-9700 CPU, with 32 GB of memory, and an NVidia GeForce GTX 1660 SUPER GPU with 6 GB.

Model training uses a properly labeled orthophoto dataset, applying a loss function based on cross entropy and the Adam optimizer for refinement. To ensure replicability and preservation of progress, we store the weights of the best-performing model in the validation set.

This practice allows for a consistent assessment of segmentation accuracy; we use the intersection over union (IoU) metric, defined as

(2) $$IoU=\frac{AreaofIntersection}{GroundTruthArea+PredictedBoxArea-AreaofIntersection}$$

where the Intersection Area represents the overlap between the model prediction and the true label, and the Ground Truth Area is the actual area of *Opuntia* spp. vegetation marked on the images. Finally, the Predicted Box Area corresponds to the area of *Opuntia* spp. vegetation that the model identifies in the image.

Furthermore, the root mean square error (RMSE) is used to assess the precision of the estimates of the vegetation area. The metric reflects the discrepancies between the observed values and those predicted by the model, defined as

(3) $$RMSE=\sqrt{\frac{1}{n}\sum_{i=1}^{n}{\left(y_{i}-{\hat{y}}_{i}\right)}^{2}}$$

where *n* is the total number of observations, $y_{i}$ represents the observed value, and $\hat{y_{i}}$ denotes the value predicted by the model for the ith observation.

The metrics used provide a quantitative basis to evaluate the ability of deep learning models to accurately segment and quantify areas of *Opuntia* spp. vegetation, facilitating comparison between different models and underscoring the effectiveness of deep learning techniques in precision agriculture.

## 4. Results

### 4.1. Comparison of Semantic Segmentation Architectures

The study performs a detailed evaluation of three architectures—UNet, DeepLabV3+, and UNet Style Xception—with a specific focus on the *Opuntia* spp. semantic segmentation task. Performance measurement is performed using RMSE and IoU metrics, and each architecture trains multiple models to obtain a robust evaluation to ensure a complete and reliable understanding of its performance. Of the three models evaluated, UNet Style Xception is the most effective in accurately identifying *Opuntia* spp. plants based on the metrics presented in Table 2. The results offer valuable guidance for the informed selection of the most appropriate architecture in contexts that require precise semantic segmentation for *Opuntia* spp. identification.

One consideration in evaluating semantic segmentation architectures is analyzing computation times on GPU and CPU, as shown in Table 2. Computation times indicate each model’s operational efficiency, with significant implications for its practical application in real-time scenarios and limited computing environments. In this study, it is observed that UNet Style Xception not only excels in accuracy, as indicated by the RMSE (1696.5831) and IoU (0.6733) metrics, but it also excels in efficiency, exhibiting the lowest processing times on GPU (0.03038 s) and CPU (0.06013 s). The comparison details that, while DeepLabV3+ and UNet offer competitive times, the optimization of UNet Style Xception positions it as the preferred architecture for contexts that demand high precision and operational efficiency.

**Figure 9:**
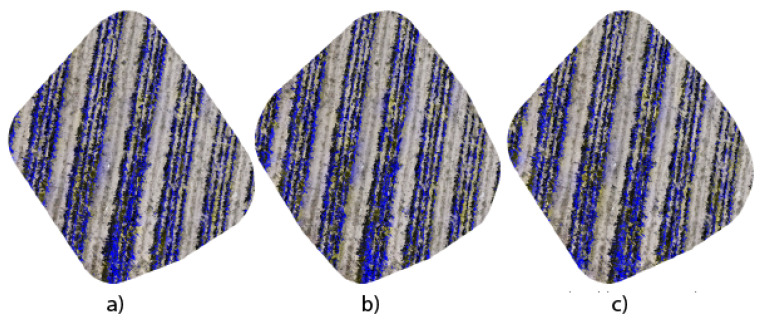
Segmentation in orthomosaics. (**a**) UNet; (**b**) DeepLabV3+; (**c**) UNet Style Xception.

Figure 9 shows the orthomosaics generated from segmented images using the three architectures proposed in this research work. The generation of orthomosaics is carried out using the open-source WebODM platform, where the results reveal not only the ability of each architecture to capture precise details of the *Opuntia* spp. but also their uniqueness in spatial representation and texture fidelity. The DeepLabV3+ application exhibits coherence in the three-dimensional reconstruction of the *Opuntia* spp., highlighting its efficiency in capturing details. On the other hand, the UNet architecture, with its layers of contraction and expansion, demonstrated the ability to preserve the topology of the *Opuntia* spp. in the resulting orthomosaic. Likewise, the UNet Style Xception variant presents an orthomosaic that combines the high precision of predictions with the ability to discern subtle nuances in the structure of the *Opuntia* spp.

**Figure 10:**
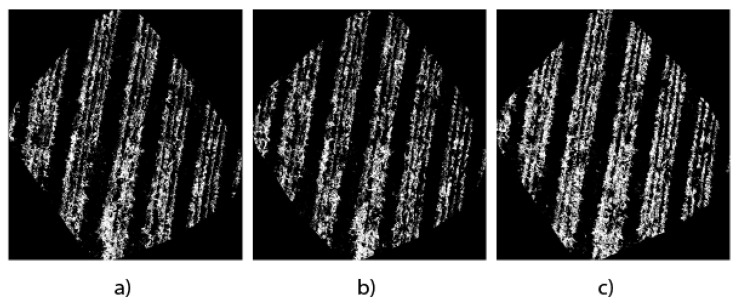
Masks obtained from each orthomosaic. (**a**) UNet; (**b**) DeepLabV3+; (**c**) UNet Style Xception.

In this work, the generation of binary masks from orthomosaics is considered very useful for the precise quantification of the plant mass of the *Opuntia* spp. cultivation because they show a general context of the study area. Figure 10 shows the results of the binary masks of the three study architectures.

The masks are the direct result of the semantic segmentation of the images, which become essential tools to carry out the pixel count and, therefore, quantify in detail the presence of *Opuntia* spp. in the region of interest to carry out a quantitative analysis and obtain approximate measurements of the *Opuntia* spp. plant cover.

### 4.2. Measurement of the Plant Mass Area of the Opuntia *spp.* Cultivation

**Figure 11:**
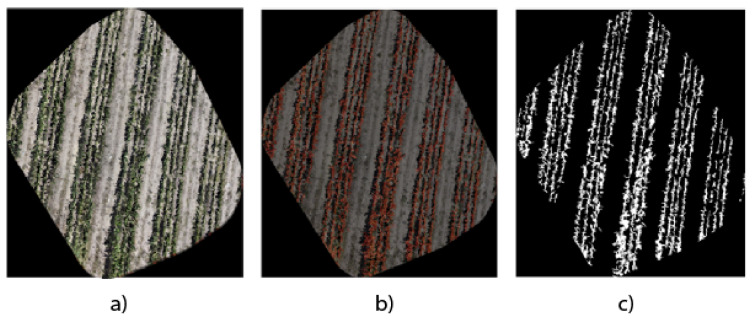
Manual segmentation. (**a**) Original orthomosaic generated in WebODM; (**b**) manual segmentation with LabelMe software; (**c**) binary mask obtained from the segmentation to quantify reference *Opuntia* spp. coverage.

The measurement from an orthomosaic is challenging because they are generally large images that exceed 4K resolution, so they cannot be introduced to a deep neural network for semantic segmentation due to storage and processing capacity issues. Therefore, we take strategies such as the one presented in the work of Gutierrez-Lazcano et al. [28], which divides the image into four and is subsequently reduced to a size of 160 pixels, which facilitates the implementation and training of different semantic segmentation models. However, it is essential to measure segmentation performance with orthomosaics since we have a general view of the crop area we want to analyze for decision making. Our analysis proposal uses the LabelMe software (Version 5.4.1) to manually segment the original orthophoto. Figure 11 shows the results of the reference segmentation from the original orthophoto image. This process allowed the generation of a detailed binary mask, essential for accurately quantifying the *Opuntia* spp. area in the region of interest.

Manual segmentation guarantees a careful and precise delimitation of the areas occupied by the *Opuntia* spp. in the orthophoto, thus providing a reliable basis for comparing the regions obtained through the three models analyzed. By using a manually segmented mask, you not only establish a reliable benchmark for evaluating automatic segmentation techniques but also enable the validation of the accuracy and effectiveness of such architectures in identification and quantification. This methodological approach provides a solid basis for comparing and critically analyzing the results obtained by automatic segmentation architectures against a manual reference standard.

The models are evaluated in detail to quantify the area of *Opuntia* spp. vegetation in each orthomosaic. Table 3 presents the quantitative results, where a significant agreement between the automatic measurements and manual quantification is evident. However, the UNet Style Xception architecture stands out notably, as it exhibited greater precision in the delimitation and quantification of *Opuntia* spp. areas compared to the other two architectures. This finding suggests adaptability and generalization capacity in the semantic segmentation task of *Opuntia* spp. vegetation.

In the existing literature [33,34,35,36,37,38], various semantic segmentation models based on deep learning have been applied to identify vegetation in aerial images. However, these studies are generally limited to smaller images and must address large orthomosaics segmentation. Our approach, instead, introduces an innovative methodology that allows the accurate and efficient segmentation of large orthomosaics, which is crucial for the extensive management of crops such as *Opuntia* spp. Table 4 compares recent approaches to segmenting vegetation areas, highlighting the resolution of the segmented images and their architectures, showing no high-resolution segmented images.

### 4.3. Discussion of Results

The results obtained show the potential that deep learning architectures possesses, specifically DeepLabV3+, UNet, and UNet Style Xception, for semantic segmentation and quantification of vegetative areas in *Opuntia* spp. cultivation. Through comparative analysis, this work highlights the significant advantages of these technologies over traditional manual methods, offering a more precise and efficient approach. However, selecting the optimal model depends on several factors, including desired accuracy and resource limitations; while UNet is distinguished by its ability to segment fine details, DeepLabV3+ and UNet Style Xception demonstrate greater adaptability to scale variations. Despite their strengths, these architectures face challenges due to the resolution imagery, application context specificity, and operational complexity.

The comparative analysis of the orthomosaics generated by the UNet, DeepLabV3+, and UNet Style Xception architectures, presented in Figure 9a–c, allows us to have a visual reference in terms of detail and edge accuracy. Meanwhile, Figure 10a–c use binary masks to quantify the vegetation area. The precision of UNet Style Xception is reflected in a higher agreement with the manual segmentation presented in Figure 11b and the binary mask in Figure 11c, showing that the UNet Style Xception architecture results are very close to the manual segmentation. The analysis highlights the ability of the UNet Style Xception architecture to handle large images and achieve accurate results.

The comparative analysis of the performance of the trained models of UNet, DeepLabV3+, and UNet Style Xception architectures for image segmentation is presented in Table 2, the IoU (>0.5) and RMSE values in a homogeneous range allow us to establish that the three models can effectively segment small images with variations in performance and execution times. The best results were from the UNet Style Xception architecture.

Table 3 presents the results of the comparative analysis of the performance of the trained models of the UNet, DeepLabV3+, and UNet Style Xception architectures for the quantification of the vegetation area in the orthomosaic. The RMSE values of the three architectures are homogeneous and allow us to establish that the three models achieve the automatic quantification of vegetation area, while the value IoU = 0.5051 and greater than 0.5, allows us to recognize that the UNet Style Xception model has the best performance for this task.

The study analyzes the architectures of DeepLabV3+, UNet standard, and UNet Style Xception. Although these methods showed competitive performance, the UNet Style Xception architecture excelled in accuracy and computational efficiency. The quantitative results presented in Table 2 indicate that UNet Style Xception obtained a lower RMSE (1696.5831) and a higher IoU (0.6733), demonstrating its robustness in handling fine details and complex structures within the vegetation. Furthermore, it presented the lowest processing times on GPU (0.03038s) and CPU (0.06013s), demonstrating its superiority in the specific *Opuntia* spp. segmentation task.

The superior performance of the UNet Style Xception architecture in this study can be attributed to its integration of separable convolutions (SeparableConv), which improve efficiency and accuracy. SeparableConv operators decompose a standard convolution into two simpler operations: a spatial convolution and a depth convolution, significantly reducing the number of calculations required and allowing faster processing without compromising accuracy [31]. This additional efficiency is essential in segmenting vegetation areas, where it is crucial to distinguish between different plant textures and details. This results in better detection and segmentation of complex structures within orthomosaic images, facilitating greater precision in the delimitation of vegetative areas.

A significant limitation of the proposed method is its dependence on the resolution and quality of the captured images. The accuracy of semantic segmentation can be negatively affected by low-resolution images, complicating the identification and quantification of small vegetative areas due to the loss of fine details that are essential for segmentation accuracy. Models such as UNet, DeepLabV3+, and UNet Style Xception present variations in their performance depending on the resolution of the images; UNet stands out for its ability to segment fine details, while DeepLabV3+ and UNet Style the environmental and climatic conditions can significantly affect the results of semantic segmentation and quantification of vegetative areas. The variability in lighting conditions and the presence of shadows represent a challenge for the generalization of the models. The air flights were scheduled around 11:00 a.m. to ensure stable lighting conditions and minimize the impact of shadows on images. However, the adaptability of the proposed method to different agricultural conditions is a key strength that provides reassurance for its potential in precision agriculture.

Additionally, weather conditions, such as cloud cover and wind, can influence the quality and clarity of captured images. To mitigate these effects, flight plans were designed with 75% lateral overlap and 70% vertical overlap, ensuring exhaustive coverage of the study area. Furthermore, the proposed approach of validating the results with orthomosaics, not only with images, allows for correcting possible errors caused by non-ideal capture conditions, providing a reliable basis for comparing and analyzing the segmentation models. Although these models have proven effective in the specific region studied, their applicability to other agricultural conditions requires additional validation. Computational efficiency is another crucial significant consideration when scaling to larger datasets. Advanced data augmentation and processing techniques are essential to maintaining data integrity and balancing computational efficiency with the quality of results. Furthermore, it is necessary to consider the complexity and operational costs to implement the proposal on a larger scale in precision agriculture. Validation using orthomosaics is essential to confirm the accuracy of the segmentation and correct possible errors caused by non-ideal capture conditions.

Furthermore, despite this study’s relatively small dataset, several strategies have been implemented to mitigate bias and enhance the model’s generalization ability. The data augmentation scheme, which includes a wide range of transformations such as flips and rotations, has significantly increased the diversity of the training images, thereby bolstering the robustness of the model. Moreover, detailed manual segmentation of the original orthophotos has been used as a reference to validate the accuracy of the automatic models, providing a solid basis for evaluating the effectiveness of semantic segmentation techniques. These combined approaches ensure that the results are consistent and reproducible while improving the model’s ability to generalize under previously unseen conditions.

On the other hand, future research can explore other advanced segmentation models, such as Segment Anything, an emerging methodology in the field of segmentation that promises greater adaptability and accuracy in various segmentation tasks. This methodology is based on the ability to segment any object in an image without needing to train a specific model for each object class; this can be advantageous in agricultural contexts where the variability of vegetative characteristics can be high. The flexibility and generalizability of Segment Anything could provide a robust framework for orthomosaic segmentation in diverse agricultural settings, potentially offering improvements in the accuracy and efficiency of the segmentation process.

## 5. Conclusions

In the present study, the semantic segmentation architectures UNet, DeepLabV3+, and UNet Style Xception are evaluated and applied to measuring areas of *Opuntia* spp. vegetation through orthomosaics. The research highlights the superiority of UNet Style Xception in terms of precision, adaptability, and generalization capacity for the delimitation and quantification of *Opuntia* spp. areas, underlining the relevance of choosing the most appropriate architecture according to the specific needs of precision and operational efficiency.

The results indicate that, although DeepLabV3+ and UNet have competitive processing times, UNet Style Xception stands out for its notable optimization. This aspect is essential in applications that demand high precision and speed, which are essential elements within the framework of precision agriculture. Furthermore, the study evidences the superiority of deep learning techniques compared to traditional manual methods in managing *Opuntia* spp. cultivation, although it also recognizes the inherent operational and economic challenges. Therefore, the need to develop pragmatic solutions to implement precision agricultural practices effectively is highlighted.

Significant progress is evident toward incorporating innovative solutions based on deep learning to manage *Opuntia* spp. cultivation. Multidisciplinary collaboration and a focus on resource optimization are key to overcoming barriers and maximizing the impact of these technologies in precision agriculture, guiding toward the sustainable management of *Opuntia* spp. crop.

## Figures and Tables

**Table 1:** Different configurations of flights, total images captured, and division of sets to evaluate the *Opuntia* spp. segmentation models.

Angle	Images	Captured	Training	Test
90°	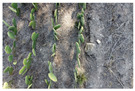	147	98	49
45°	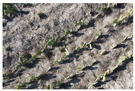	150	98	52
135°	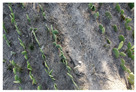	146	98	48
	Total	443	294	149

**Table 2:** Comparative performance analysis of UNet, DeepLabV3+, and UNet Style Xception architectures for image segmentation.

Architecture	RMSE	IoU	Time GPU	Time CPU
UNet	1758.0079	0.6657	0.04242 s	0.12059 s
DeepLab3+	1779.3001	0.6562	0.03733 s	0.06073 s
UNet Style Xception	1696.5831	0.6733	0.03038 s	0.06013 s

**Table 3:** Comparative performance analysis of UNet, DeepLabV3+, and UNet Style Xception architectures for vegetation area quantification in orthomosaics.

Architecture	Pixel Count	Vegetation (m^2^)	IoU	RMSE
UNet	127,536	53.5970 m^2^	0.3106	89.2447
DeepLabV3+	124,451	52.3005 m^2^	0.4755	77.6231
UNet Style Xception	128,194	53.8735 m^2^	0.5051	83.1994
Reference	128,959	54.1950 m^2^	1.00	0.00

**Table 4:** Comparison of recent approaches in segmenting vegetation areas using semantic segmentation and deep learning architectures.

Proposal	Approach	Method	Metrics	Segmented Image Size (px)	Orthomosaics	Limitations
Sa et al. [33]	Large-scale weed mapping in precision agriculture	Deep Neural Network SegNet	Precision and Recall	480 × 360	Yes	Reliable spatio-temporal models for complex plants and fields
Ayhan et al. [34]	Compare deep and conventional methods	DeepLabV3+ and custom CNN	IoU and RMSE	512 × 512	No	Large data, image differences, channel restrictions, imbalance
Zhang et al. [35]	Multi-scale context aggregation	HRNet	Precision and F1	512 × 512	No	Computational needs, scalability, generalization validation
Lan et al. [36]	Real-time weed identification	U-Net, MobileNetV2-UNet, BiSeNetV2 and FFB-BiSeNetV2	Accuracy and IoU	650 × 800	No	Data, variability, channels, resources
Xiao et al. [37]	Segmentation adaptation to different domains	WildNet	IoU, F1 Score	512 × 512 and 896 × 896	No	Dataset dependency, computational needs, generalization, complexity
Zhu et al. [38]	Handling temporal sequences	xLSTM	Accuracy and IoU	512 × 512	No	Subpar performance, unidirectional scanning, data requirements, computational complexity

## Data Availability

Data are contained within the article.

## References

[1] Resolution G.A. (2015). Transforming our World: The 2030 Agenda for Sustainable Development. UN Doc. A/RES/70/1. https://sustainabledevelopment.un.org/post2015/transformingourworld/publication.

[2] Sáenz C., Berger H. (2006). Utilización Agroindustrial del Nopal.

[3] Díaz M.d.S.S., de La Rosa A.-P.B., Héliès-Toussaint C., Guéraud F., Nègre-Salvayre A. (2017). *Opuntia* spp.: Characterization and benefits in chronic diseases. Oxidative Med. Cell. Longev..

[4] Louhaichi M., Nefzaoui A., Guevara J.C. (2017). Cactus Ecosystem Goods and Services.

[5] Servicio de Administración Agroalimentaria y Pesquera (SIAP) (2023). Panorama Agroalimentario 2023.

[6] Zhang N., Wang M., Wang N. (2002). Precision agriculture—A worldwide overview. Comput. Electron. Agric..

[7] Crusiol L.G.T., Sun L., Sun Z., Chen R., Wu Y., Ma J., Song C. (2022). In-Season Monitoring of Maize Leaf Water Content Using Ground-Based and UAV-Based Hyperspectral Data. Sustainability.

[8] Giovos R., Tassopoulos D., Kalivas D., Lougkos N., Priovolou A. (2021). Remote sensing vegetation indices in viticulture: A critical review. Agriculture.

[9] Messina G., Modica G. (2022). The role of remote sensing in olive growing farm management: A research outlook from 2000 to the present in the framework of precision agriculture applications. Remote Sens..

[10] Candiago S., Remondino F., De Giglio M., Dubbini M., Gattelli M. (2015). Evaluating multispectral images and vegetation indices for precision farming applications from UAV images. Remote Sens..

[11] Radočaj D., Jurišić M., Gašparović M. (2022). The role of remote sensing data and methods in a modern approach to fertilization in precision agriculture. Remote Sens..

[12] Pascucci S., Pignatti S., Casa R., Darvishzadeh R., Huang W. (2020). Special issue “hyperspectral remote sensing of agriculture and vegetation”. Remote Sens..

[13] Ennouri K., Smaoui S., Gharbi Y., Cheffi M., Ben Braiek O., Ennouri M., Triki M.A. (2021). Usage of artificial intelligence and remote sensing as efficient devices to increase agricultural system yields. J. Food Qual..

[14] Lee C.-J., Yang M.-D., Tseng H.-H., Hsu Y.-C., Sung Y., Chen W.-L. (2023). Single-plant broccoli growth monitoring using deep learning with UAV imagery. Comput. Electron. Agric..

[15] Lillesand T.M., Kiefer R.W., Chipman J.W. (2015). Remote Sensing and Image Interpretation.

[16] Pal M., Foody G.M. (2010). Feature selection for classification of hyperspectral data by SVM. IEEE Trans. Geosci. Remote Sens..

[17] Torres-Sánchez J., Peña J.M., de Castro A.I., López-Granados F. (2014). Multi-temporal mapping of the vegetation fraction in early-season wheat fields using images from UAV. Comput. Electron. Agric..

[18] LeCun Y., Bengio Y., Hinton G. (2015). Deep learning. Nature.

[19] Krizhevsky A., Sutskever I., Hinton G.E. (2017). ImageNet classification with deep convolutional neural networks. Commun. ACM.

[20] Kamilaris A., Prenafeta-Boldú F.X. (2018). Deep learning in agriculture: A survey. Comput. Electron. Agric..

[21] Milioto A., Lottes P., Stachniss C. Real-time semantic segmentation of crop and weed for precision agriculture robots leveraging background knowledge in CNNs. Proceedings of the 2018 IEEE International Conference on Robotics and Automation (ICRA).

[22] Li Y., Liu Z., Lai Q., Li S., Guo Y., Wang Y., Dai Z., Huang J. (2022). ESA-UNet for assisted diagnosis of cardiac magnetic resonance image based on the semantic segmentation of the heart. Front. Cardiovasc. Med..

[23] Rehman A., Saba T., Kashif M., Fati S.M., Bahaj S.A., Chaudhry H. (2022). A revisit of internet of things technologies for monitoring and control strategies in smart agriculture. Agronomy.

[24] Chen L.-C., Zhu Y., Papandreou G., Schroff F., Adam H., Ferrari V., Hebert M., Sminchisescu C., Weiss Y. (2018). Encoder-Decoder with Atrous Separable Convolution for Semantic Image Segmentation. Computer Vision—ECCV 2018.

[25] Ronneberger O., Fischer P., Brox T., Navab N., Hornegger J., Wells W.M., Frangi A.F. (2015). U-Net: Convolutional Networks for Biomedical Image Segmentation. Medical Image Computing and Computer-Assisted Intervention—MICCAI 2015.

[26] Chollet F. Image Segmentation with a U-Net-Like Architecture. https://keras.io/examples/vision/oxford_pets_image_segmentation/.

[27] Shahid R., Qureshi W.S., Khan U.S., Munir A., Zeb A., Moazzam S.I. (2024). Aerial imagery-based tobacco plant counting framework for efficient crop emergence estimation. Comput. Electron. Agric..

[28] Gutiérrez-Lazcano L., Camacho-Bello C.J., Cornejo-Velazquez E., Arroyo-Núñez J.H., Clavel-Maqueda M. (2022). *Cuscuta* spp. Segmentation Based on Unmanned Aerial Vehicles (UAVs) and Orthomosaics Using a U-Net Xception-Style Model. Remote Sens..

[29] Mario D.M. (1981). Cultivo de Nopal de Verdura.

[30] Reyes-Terrazas A.S., Flores-Sánchez D., Navarro-Garza H., Pérez-Olvera M.A., Almaguer-Vargas G. (2023). Características y retos del sistema de cultivo de nopal verdura en Cuautlancingo, Otumba. Rev. Mex. Cienc. Agrícolas.

[31] Chollet F. Xception: Deep Learning with Depthwise Separable Convolutions. Proceedings of the 2017 IEEE Conference on Computer Vision and Pattern Recognition (CVPR).

[32] OpenDroneMap Community (2022). WebODM: User-Friendly, Commercial-Grade Software for Drone Image Processing. https://www.webodm.org/.

[33] Sa I., Popović M., Khanna R., Chen Z., Lottes P., Liebisch F., Nieto J., Stachniss C., Walter A., Siegwart R. (2018). WeedMap: A large-scale semantic weed mapping framework using aerial multispectral imaging and deep neural network for precision farming. Remote Sens..

[34] Ayhan B., Kwan C., Budavari B., Kwan L., Lu Y., Perez D., Li J., Skarlatos D., Vlachos M. (2020). Vegetation detection using deep learning and conventional methods. Remote Sens..

[35] Zhang J., Lin S., Ding L., Bruzzone L. (2020). Multi-scale context aggregation for semantic segmentation of remote sensing images. Remote Sens..

[36] Lan Y., Huang K., Yang C., Lei L., Ye J., Zhang J., Zeng W., Zhang Y., Deng J. (2021). Real-time identification of rice weeds by UAV low-altitude remote sensing based on improved semantic segmentation model. Remote Sens..

[37] Xiao H., Yao W., Chen H., Cheng L., Li B., Ren L. (2023). SCDA: A Style and Content Domain Adaptive Semantic Segmentation Method for Remote Sensing Images. Remote Sens..

[38] Zhu Q., Cai Y., Fan L. (2024). Seg-LSTM: Performance of xLSTM for Semantic Segmentation of Remotely Sensed Images. arXiv.

